# Stress Concentration of Hybrid Occlusal Splint-Mouthguard during a Simulated Maxillofacial Traumatic Impact: 3D-FEA

**DOI:** 10.3390/dj10040065

**Published:** 2022-04-06

**Authors:** João Paulo Mendes Tribst, Amanda Maria de Oliveira Dal Piva, Les Kalman

**Affiliations:** 1 Department of Dental Materials, Academic Centre for Dentistry Amsterdam (ACTA), University of Amsterdam and Vrije Universiteit Amsterdam, 1081 LA Amsterdam, The Netherlands; joao.tribst@gmail.com; 2 Schulich School of Medicine & Dentistry, Western University, 1151 Richmond St., London, ON N6A 3K7, Canada; lkalman@uwo.ca

**Keywords:** mouthguard, occlusal splint, trauma, finite element analysis, athletic injuries

## Abstract

Mouthguards (MG) are protective devices that can reduce the risks of facial trauma. However, many athletes do not use them. Additionally, MG wear with coincidental parafunctional activity has not been considered. The aim of this study was to evaluate the stress distribution as a consequence of a direct impact comparing a conventional MG with a novel hybrid appliance (HMG). Using computer-aided design (CAD) software, a human skull was modeled with the teeth inserted into their respective alveolus. The models were divided according to the MG type (conventional or hybrid). The geometries were exported to the computer-aided engineering (CAE) software and the materials were considered isotropic. Fixation was defined at the base of the maxilla. The load was applied using a hockey puck. The total deformation (mm) and the von Mises stress (MPa) results were obtained for the MGs (conventional and hybrid), upper teeth, lower teeth, and maxillary bone. Despite the presence of an MG, it is still possible to observe generated stress in all structures. However, the hybrid design was more efficient than the conventional design in reducing the displacement during the impact and consequently the stress on the upper teeth, lower teeth, and maxillary bone. Higher stress magnitude was more concentrated at the inner portion of the hybrid design than the conventional device. The HMG appliance decreased the stress concentration in the teeth and in the bone, limiting the areas susceptible to injuries to the regions directly impacted by the hockey puck. Although the novel HMG may mitigate injury, some stress will still result, and any possible injury should be evaluated by a dental professional.

## 1. Introduction

Despite all the protective devices that hockey players wear, they are not completely safe from injury. Hockey (ice or field) is considered one of the most dangerous sports, due to the skate blades, the puck or hockey balls that travel at rapid speed, hockey sticks themselves, and contact with another opponent [1,2,3,4,5]. Therefore, many injuries, especially maxillofacial ones, are reported in this sport. Many of the dental injuries may be restored through dentistry, but like all rehabilitations, the repair will have a limited lifespan and may be an issue to athletic performance.

Dentofacial trauma represents a serious problem, and a considerable number of athletes still do not regularly wear mouthguards. The likelihood is that if mouthguard usage were higher, fewer dentofacial injuries would occur during games and training [6].

To appropriately protect teeth, bones, and the temporomandibular joint from damage during contact activities and sports, athletes should wear a mouthguard (MG) appliance [1,2,3,4]. MGs may be stock (over the counter), boil and bite, and custom fabricated [5,7]. The latter is considered more appropriate due to its greater protective capacity compared to the other two [7]. Custom MGs are patient-specific and allow for better stress reduction, retention, comfort and fit, with only minor impacts on speech and breathing [5]. It has been reported that MGs in vinyl acetate copolymer (EVA) at a thickness of 4 mm can improve the stress dissipation, offering maximal protection of the teeth and adjacent facial bone structures [7,8], since flexible polymers are able to distribute the stress concentration during a traumatic impact [9,10]. In the Netherlands, the Royal Dutch Hockey Association stated in 2015 that all field hockey players should use an MG during all activities that occur on the field [11]. The authors investigated this new Dutch regulation and assessed the number and severity of orofacial injuries reporting that the severity and frequency of the injuries trended downwards after the mandatory MG use protocol [12].

Another relevant concern is about patients with some form of parafunction. For example, in cases of patients with bruxism, one of the most common approaches is the use of a rigid occlusal splint [13], which will protect the teeth and may affect the occlusion and maxillofacial musculature [14,15].

Recently, a hybrid MG design has been developed with a higher number of occlusal contacts, minimal vertical dimension change and condylar displacement, in comparison with the other types of MGs [16,17]. This novel MG has an occlusal splint combined with flexible axial flanges [5]. This hybrid occlusal splint-mouthguard (HMG) was beneficial in reducing the jaw displacement during chewing and the generated stresses in upper and lower teeth, when a compressive loading has been evaluated [5]. At the same time, the presence of a rigid occlusal splint, to stabilize the occlusion, has been advocated as a promising alternative to conventional MGs for patients with bruxism [14]. However, there is lack of data regarding this HMG design during traumatic impact.

With the aid of digital dentistry, a virtual design of a multilayer MG can be performed within two hours [17]. However, it requires certain modelling skills and financial investments in the freeform software [17]. Conventionally, multilayer custom MGs can be manufactured using the analog method as applied to manufacture the present HMG concept.

It has been documented that the use of MGs promotes greater orofacial protection for athletes who play sports with some risk of injury in this region [1]. To demonstrate this, many three-dimensional (3D) numerical simulations were developed to show the protection provided by the proper use of an MG against injuries to the teeth and facial bones [3,4,5,6,7,17,18]. These 3D investigations utilize finite element analysis (FEA) and allow qualitative and quantitative evaluation of the effects of using an MG, illustrating the stresses/strain and also their distribution during a traumatic impact [7,18]. FEA has controlled conditions, without the need to harm a patient or animal, to evaluate the impact on the skull [19]. It is therefore considered to be one of the most suitable methodologies [20,21] for this purpose. There have been no studies to investigate the benefits of using the new hybrid MG in patients who practice contact sports. Therefore, the aim of this study was to analyze the biomechanical effects of wearing a novel HMG on the mechanical response of the teeth, bone, and the appliance itself, when subjected to a simulated hockey puck impact.

## 2. Materials and Methods

The mechanical analysis was performed with the finite element analysis (FEA) and a computer-aided engineering software (ANSYS 19.2; ANSYS Inc., Houston, TX, USA).

**Figure 1:**
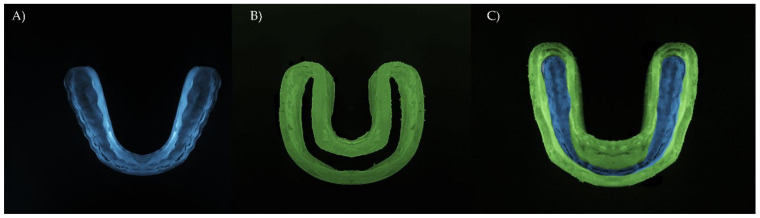
Hybrid occlusal splint mouthguard design (Kalman et al., 2022 [5]). (**A**) Polycarbonate as a rigid inner portion, (**B**) ethylene vinyl acetate used in the axial flexible flanges, and (**C**) the final modulated appliance assembled as the novel HMG.

For the three-dimensional (3D) modeling, a skull model has been imported from previous studies [5,7] and the 3D volumes simulating bones, teeth, and supporting tissues were individualized using the computer aided design (CAD) software (Rhinoceros version 4.0 SR8; McNeel North America, Seattle, WA, USA). In the same way, the hybrid MG model has been imported from another study that has digitalized with its two distinct and juxtaposed structures: a rigid occlusal splint and axial flanges made of 3 mm flexible polymer (Figure 1) [5]. After, the STL files were converted to volumetric solids using the reverse engineering tool and BioCAD protocol [20].

**Figure 2:**
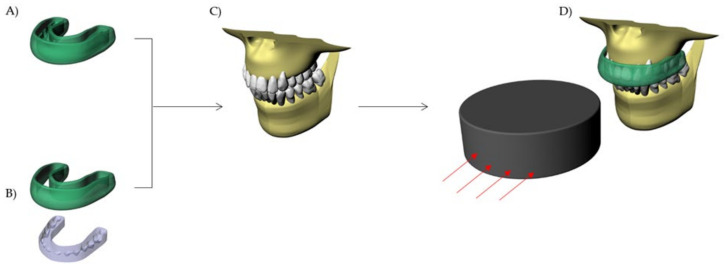
Schematic illustration of the evaluated MGs: (**A**) Conventional custom-made mouthguard; and (**B**) Hybrid mouthguard (HMG) device with combined occlusal splint and flexible axial flanges. (**C**) Maxillary model without MG and (**D**) MG in position with hockey puck impact direction.

In this study, there was the simulation of a puck impacting the maxillofacial region of an athlete using both hybrid and conventional MG. Therefore, as the control group, a conventional custom-made MG [7,8] was simulated with 4 mm thickness [20]. A Boolean difference was considered for both MGs to create the inner portion with occlusal impression (Figure 2).

**Figure 3:**
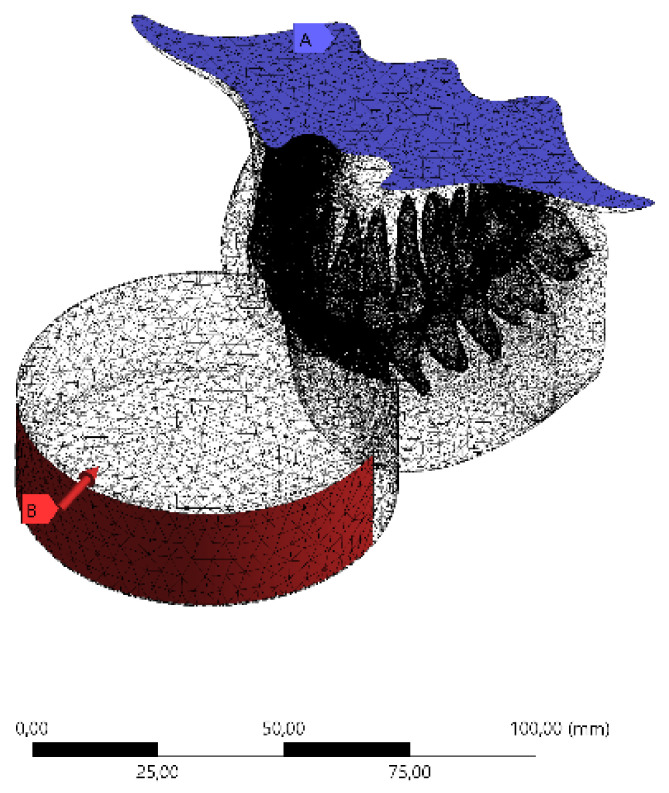
Boundary conditions in the 3D finite element model: Meshing subdivision and fixed support with impact path restricted to the *y*-axis.

The models were exported to a computer aided engineering (CAE) software (ANSYS 19.2, ANSYS Inc., Houston, TX, USA) in a Standard for the Exchange of Product Data (STEP) format. In sequence, the mesh was processed through the convergence test until a finite number of nodes and elements were obtained. The puck impact was simulated using mechanical multiple step contact analysis. Boundary conditions defined the model with unrestrained occlusal path after the initial velocity was applied in the hockey puck (100 m/s) [21,22,23]. No gravitational or air-friction forces were considered. The base surface of the maxillary bone was restricted in X, Y, and Z directions [18] (Figure 3).

For the analysis, all materials and structures were stated as homogeneous, isotropic, linear, and elastic. The contacts were considered perfectly bonded with the exception of the MG that was considered frictionless [18]. In this study, the stress concentration in the MG, teeth, and upper jaw was analyzed with Total Deformation (mm) and von Mises Stress (MPa) criteria. The mechanical properties used in the simulation are summarized in Table 1.

## 3. Results and Discussion

**Figure 4:**
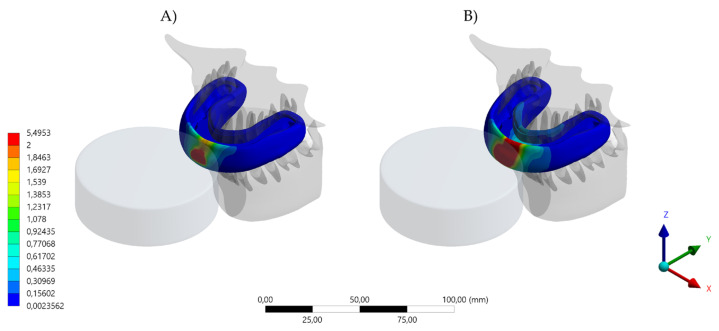
Total deformation result in the MG. (**A**) Device displacement with the conventional design and (**B**) device displacement with the hybrid (HMG) design.

**Figure 5:**
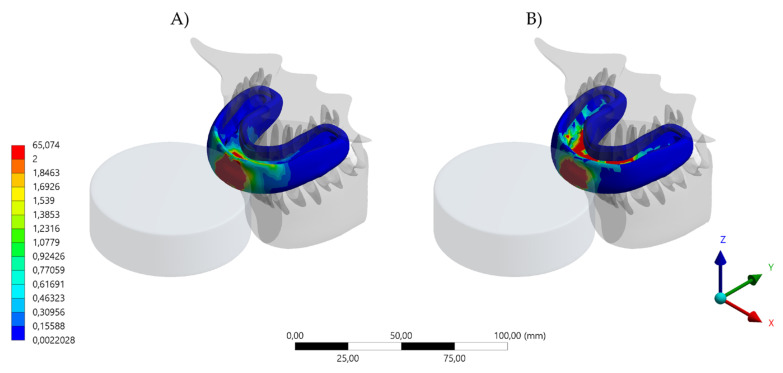
Von Mises Stress result in the MG. (**A**) Device stress with the conventional design and (**B**) device stress with the hybrid (HMG) design.

**Figure 6:**
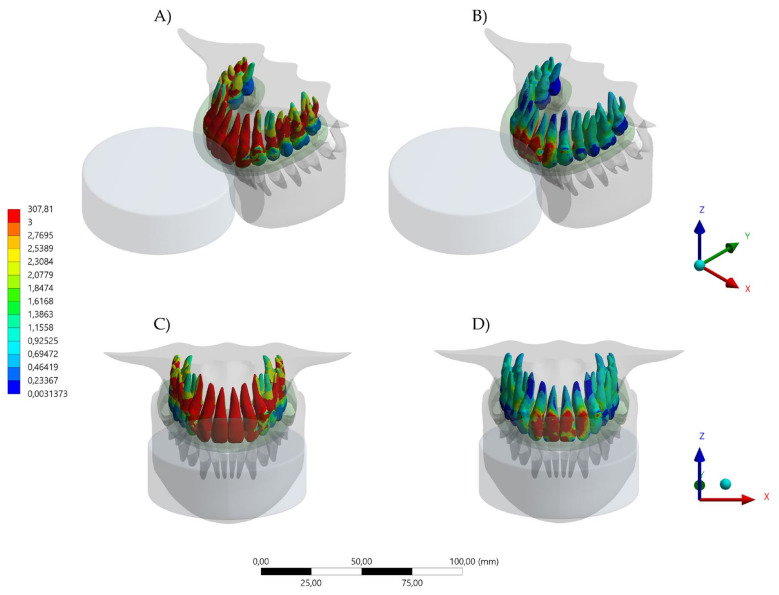
Von Mises Stress result in upper teeth. (**A**) Isometric view of the upper teeth stresss with the conventional design and (**B**) isometric view of the upper teeth stresss with the hybrid (HMG) design. (**C**) Buccal view of the upper teeth stresss with the conventional design and (**D**) buccal view of the upper teeth stresss with the hybrid (HMG) design.

**Figure 7:**
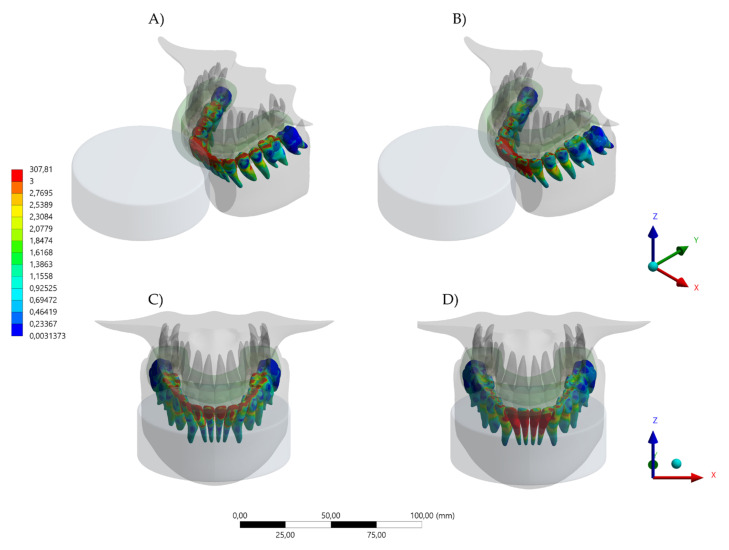
Von Mises Stress result in lower teeth. (**A**) Isometric view of the lower teeth stresss with the conventional design and (**B**) isometric view of the lower teeth stresss with the hybrid (HMG) design. (**C**) Buccal view of the lower teeth stresss with the conventional design and (**D**) buccal view of the lower teeth stresss with the hybrid (HMG) design.

**Figure 8:**
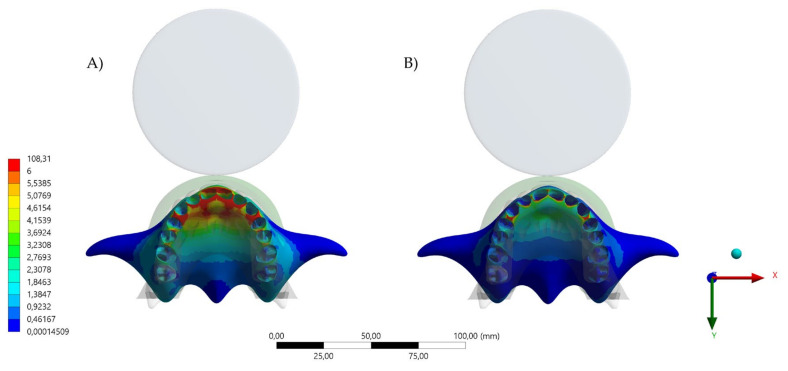
Von Mises stress result in the bone tissue. (**A**) Occlusal view of the maxillary bone stresss with the conventional design and (**B**) occlusal view of the maxillary bone stress with the hybrid (HMG) design.

The aim of this study was to investigate the protective effect of wearing a novel MG with a hybrid occlusal splint design on the mechanical response of the teeth, bone, and the device itself when subjected to simulated dental trauma. It was observed that the MG design affected the stress level in the evaluated structures. Total deformation and von Mises stress results in the MG indicated that the HMG retains more stress in the area near the teeth, while the conventional MG concentrates the stresses in the buccal face of the MG itself (Figure 4, Figure 5, Figure 6, Figure 7 and Figure 8).

Different contact activities result in various maxillofacial injuries, which could occur from falls, collisions with other players and collisions with rigid structures [23,24]. Despite an abundance of scientific literature that promotes the importance of mouthguard use as a preventive approach, many athletes do not wear an MG [3,4,5,6,7,8,9,10,11,12]. The majority of excuses for not wearing the MG have been related to discomfort, difficulty in breathing, talking, and swallowing during an activity [1,25,26]. For that reason, the present study simulated a mouthguard with the recommended thickness of 4 mm [20], aiming for a balance between comfort and protection. Despite that, the von Mises stress results indicated that the HMG device was more effective in protecting the upper teeth and decreasing the stresses concentration in comparison to the conventional MG. The areas with lower stress magnitude were the buccal face of all four incisors (Figure 6). When evaluating the antagonists, the HMG exhibited a decrease in the number of teeth with concentrated stresses; however, the incisors presented a higher stress concentration, with the possibility of increasing the risk of injury (Figure 7).

For the bone tissue, the anterior region of the upper jaw was more effectively protected by the HMG as compared to the conventional MG. This investigation is in agreement with previous reports [3,4,5,6,7,18,19,20], that reinforce the importance of properly wearing an MG during sport activities to minimize the effect of maxillofacial trauma from a mishap. In competition, athletes perform stressful workouts to achieve maximum performance. Although regular sport activities improve general health, those same activities may negatively impact the athlete’s well-being and quality of life from dental trauma, requiring immediate treatment [5]. Thereafter, properly wearing an MG appliance or device receives strong recommendation regardless of the patient’s occlusion [3]. This study assessed trauma simulation with occlusion considerations, with the antagonist teeth contacting the bottom surface of the device [27,28].

The scientific literature demonstrates numerous designs of mouthguards that can theoretically provide different types of protection, with variations in cost and fabrication approaches. However, all the appliances or devices are composed of polymeric materials and are indicated to reduce the impact energy at the moment of the traumatic accident [7,8]. In addition, the protective effect of a custom-made device is superior to stock and boil and bite appliances in reducing the stress magnitude during an impact [7]. This is due to the custom fit to the patient, with uniform thickness and symmetric alignment. However, it is important to note that during the fabrication process, the mouthguard appliance can be affected by several parameters which can reduce its fit, form, and protective function [27,29,30]. Previous three-dimensional FEA studies have demonstrated the positive effect of MG use for teeth [3,4,5,6,7], providing stress reduction for dentin, enamel [3,18,28], and bone tissue [3,18,28] at the site of impact when an MG is in position.

Approximately 31% of orofacial injuries result from sport-related trauma and 50% of those are oral/dental injuries. In athletes that practices contact sports, the prevalence of orofacial injuries is 39.1%; with variations in the injury type, sport, level of competition, participant’s age, sex, and other factors [30,31]. A study evaluating 169 ice hockey players in Canada indicated that 45.6% of the athletes never wore an MG, 23.1% always wore an MG, 14.8% sometimes wore an MG, and 16.5% only wore an MG when it was enforced [32]. In addition, 57.7% of the players have been hit by a stick, 46.2% by a puck, and 25% by an opponent [31,32,33]. This data can be complemented by the present results, showing that the stress at the alveolar bone level can be significant reduced with the use of HMG (Figure 8).

According to Hayashi [33], MGs used by field hockey players after two years of continuous use resulted in a significant reduction in MG thicknesses and a significantly increased MG length. According to the authors, MG dimensions were affected by clenching, bruxism, and others oral functions. In addition, the practicing sports can affect the oral health through dehydration, stress, bruxism (teeth grinding), tooth erosion, and abrasion [1,26,33]. According to the literature, there is a complex bilateral interaction between oral health and physical activity and that oral diseases can influence the physical exercise outcome, risk of injury, and performance [1,5,33]. The HMG, with an integrated occlusal splint, would address and alleviate many of these occlusally-driven concerns in conventional MG appliances [5].

A previous study, combining finite element method with clinical data, indicated that an occlusal splint was effective to reduce the stress in the jaw [34]. According to the authors, the parafunctional habits can be regulated with the occlusal splint with a reduction of deformation in the temporomandibular joint [34]. In a similar manner, another FEA demonstrated that a rigid occlusal splint can be effective to evenly distribute the functional load in the treatment of patients with parafunction [35,36]. It is therefore a concern for athletes that have episodes of bruxism and recurrent clenching during the practice of physical or sport activity [37]. The novel design evaluated in this investigation has been previously reported in another study that evaluated its mechanical response during compression by the jaw [5]. That study indicated that there was a beneficial effect for athletes with parafunctional habits when wearing this device [5,16]. This investigation complements those results, indicating that during a traumatic impact, the hybrid MG (HMG) reduces the stress magnitude in the upper teeth, lower teeth, and bone.

According to current recommendations for dentists [36,37,38,39], after delivering an MG to a patient, the dentist plays an important role in providing patient instructions that have a focus on motivation and proper care of the mouthguard [36,37,38,39]. Similarly, this study complements this approach, suggesting follow-up clinical appointments to ensure that occlusion, and the fit and function of the HMG, should be assessed and optimized.

Similar to the laboratorial and numerical studies, this investigation has limitations. The simulated impact was applied in just one direction; however, impacts in other regions could generate different results [3,4]. In addition, the materials were assumed isotropic, which is not the case [8]. The model was based on an adult maxilla with completely formed teeth, a perfect dentition, the absence of restorations and an ideal occlusion [17]. There was no aging effect or wear caused by the long-term use considered in the polymeric material [33]. There was no presence of oral fluids, pH variation, temperature variation or soft tissue presence [6]. Further studies, including these factors, should be performed.

## 4. Conclusions

The novel hybrid MG appliance has a design and functionality that decreased the stress concentration in the teeth and in bone when a simulated hockey puck traumatic event has occurred. Although the mouthguard may mitigate injury, some stress will still result, and any possible injury should be evaluated by a dental professional.

## Figures and Tables

**Table 1:** Mechanical properties considered in the finite element analysis.

Material/Structure	Elastic Modulus (MPa)	Poisson Ratio	Density (g/cm^3^)
Enamel	84,100	0.30	2.14
Dentin	18,600	0.30	2.97
Bone tissue	13,700	0.30	2.00
Polycarbonate	2200	0.30	1.20
Ethylene vinyl acetate	18	0.30	0.95

## Data Availability

Data available on request.
